# Potential biomarkers of ductal carcinoma in situ progression

**DOI:** 10.1186/s12885-020-6608-y

**Published:** 2020-02-12

**Authors:** Raquel Spinassé Dettogni, Elaine Stur, Ana Carolina Laus, René Aloísio da Costa Vieira, Márcia Maria Chiquitelli Marques, Iara Viana Vidigal Santana, José Zago Pulido, Laura Fregonassi Ribeiro, Narelle de Jesus Parmanhani, Lidiane Pignaton Agostini, Raquel Silva dos Reis, Eldamária de Vargas Wolfgramm dos Santos, Lyvia Neves Rebello Alves, Fernanda Mariano Garcia, Jéssica Aflávio Santos, Diego do Prado Ventorim, Rui Manuel Reis, Iúri Drumond Louro

**Affiliations:** 1 Department of Biological Sciences-Human and Molecular Genetics Nucleus, Federal University of Espirito Santo, Vitoria, Espirito Santo Brazil; 2 Molecular Oncology Research Center-Barretos Cancer Hospital, Barretos, Sao Paulo Brazil; 3 Department of Mastology and Breast Reconstruction-Barretos Cancer Hospital, Barretos, Sao Paulo Brazil; 4 Barretos School of Health Sciences-FACISB, Barretos, Sao Paulo Brazil; 5 Department of Pathology-Barretos Cancer Hospital, Barretos, Sao Paulo Brazil; 6 Evangelical Hospital of Cachoeiro de Itapemirim, Cachoeiro de Itapemirim, Espirito Santo Brazil; 7 Oncology Clinical Research Center, Cachoeiro de Itapemirim, Espirito Santo Brazil; 8 Life and Health Sciences Research Institute (ICVS)-Health Sciences School, University of Minho, Braga, Portugal; 9 ICVS/3B’s-PT Government Associate Laboratory, Braga/Guimarães, Portugal

**Keywords:** Ductal carcinoma in situ, Tumor progression, *FGF2*, *GAS1*, *SFRP1*

## Abstract

**Background:**

Ductal carcinoma in situ is a non-obligate precursor of invasive breast carcinoma and presents a potential risk of over or undertreatment. Finding molecular biomarkers of disease progression could allow for more adequate patient treatment. We aimed to identify potential biomarkers that can predict invasiveness risk.

**Methods:**

In this epithelial cell-based study archival formalin-fixed paraffin-embedded blocks from six patients diagnosed with invasive lesions (pure invasive ductal carcinoma), six with in-situ lesions (pure ductal carcinoma in situ*)*, six with synchronous lesions (invasive ductal carcinoma with an in-situ component) and three non-neoplastic breast epithelium tissues were analyzed by gene expression profiling of 770 genes, using the *nCounter® PanCancer Pathways panel* of NanoString Technologies.

**Results:**

The results showed that in comparison with non-neoplastic tissue the pure ductal carcinoma in situ was one with the most altered gene expression profile. Comparing pure ductal carcinoma in situ and in-situ component six differentially expressed genes were found, three of them (*FGF2*, *GAS1,* and *SFRP1*), play a role in cell invasiveness. Importantly, these genes were also differentially expressed between invasive and noninvasive groups and were negatively regulated in later stages of carcinogenesis.

**Conclusions:**

We propose these three genes (*FGF2*, *GAS1,* and *SFRP1*) as potential biomarkers of ductal carcinoma in situ progression, suggesting that their downregulation may be involved in the transition of stationary to migrating invasive epithelial cells.

## Background

Breast cancer (BC) begins as premalignant lesions, progressing to the preinvasive stage of ductal carcinoma in situ (DCIS) and culminating as invasive ductal carcinoma (IDC) [1, 2]. DCIS represents 20–25% of newly diagnosed BC and up to 40% can progress to IDC [3]. Gene expression profiling-based studies have shown that distinct stages of progression are evolutionary products of same clonal origin and that genes conferring invasive growth are disrupted during preinvasive stages [4–8]. Differences among these stages are not clear and there is no consensus as to how gene activation or inactivation alters the course of BC progression.

DCIS is a form of BC where epithelial cells restricted to the ducts exhibit an atypical phenotype [8]. Interestingly, some DCIS lesions progress to IDCs, while others remain unchanged [9]. Finding gene expression patterns that could predict invasive progression would allow us to personalize DCIS treatment to each patient’s real needs.

In this study, gene expression profiling was performed in non-neoplastic breast epithelium, pure DCIS, mixed lesions (DCIS-IDC) (IDC with an in-situ component) and pure IDCs, aiming to identify molecular predictors of invasive disease risk.

## Materials and methods

### Study population

Formalin-fixed paraffin-embedded (FFPE) breast blocks of 3 healthy women were selected as non-neoplastic breast epithelium. Specimens with pathological lesions (IDC, DCIS, DCIS-IDC) were obtained from the Department of Pathology of Barretos Cancer Hospital-Sao Paulo, Brazil. Archival FFPE blocks from 6 patients diagnosed with IDC, 6 with DCIS and 6 with IDC with in-situ (DCIS-IDC) component were selected (Table 1). Cases of IDC and DCIS-IDC were chosen considering the molecular subtype, according to St. Gallen consensus [13]. Pathological staging was defined by current edition in 2015 of TNM classification [10]. Histological grade was determined as Lakhani et al. [12]. Myriad’s hereditary cancer tests were done by Myriad Genetic Laboratories, Inc. (*Salt Lake City, Utah, USA)* through observations of deleterious mutations, as published by Frank et al. [11]. Selected patients had a mean age of 55 years and were not under risk of hereditary BC, they did not present metastasis and did not receive any treatment prior surgery.

**Table 1:** Patients characteristics

Case ID	Age range (years)	Invasive type	Molecular subtype	Histological type	Histological grade	Invasive nuclear grade	DCIS Nuclear grade	DCIS type	ER^f^ in DCIS	PR^g^ in DCIS	Size (mm)	T^h^ stage	N^i^ stage	Pathological clinical stage	Myriad test
G1P1	50–60	IDC^a^	Luminal B Her negativo	IDC	3	3	N	N	N	N	31	T2	N0	IIa	*N* > 50
G1P2	50–60	IDC	TN^d^	IDC	3	3	N	N	N	N	10	T1b	N0	I	*N* > 50
G1P3	60–70	IDC	HER2	IDC	3	3	N	N	N	N	33	T2	N1	IIb	*N* > 50
G1P4	60–70	IDC	Luminal A	IDC	2	2	N	N	N	N	20	T1c	N0	I	*N* > 50
G1P5	40–50	IDC	Luminal B Her positivo	IDC	1	1	N	N	N	N	40	T2	N0	IIa	*N* < 50
G1P6	40–50	IDC	Luminal B Her negativo	IDC	2	2	N	N	N	N	20	T1c	N0	I	*N* < 50
G2P1	70–80	N^b^	N	DCIS^e^	N	N	3	S, C, M, Co	++++	+++	105	Tis	N0	0	*N* > 50
G2P2	40–50	N	N	DCIS	N	N	3	S, C, M	–	–	50	Tis	N0	0	*N* < 50
G2P3	40–50	N	N	DCIS	N	N	3	S, Co, C, M	+++	+++	18	Tis	N0	0	*N* < 50
G2P4	50–60	N	N	DCIS	N	N	3	S, Co	++++	++	30	Tis	N0	0	*N* > 50
G2P5	50–60	N	N	DCIS	N	N	3	C, M, Co	+	+	60	Tis	N0	0	*N* > 50
G2P6	50–60	N	N	DCIS	N	N	3	S, M, Co	+	–	20	Tis	N0	0	*N* > 50
G3P1	50–60	^*^IDC^c^	TN	DCIS-IDC	3	3	3	S, C, Co	–	–	30	T1c	N0	I	*N* > 50
G3P2	60–70	^*^IDC	Her2	DCIS-IDC	3	3	3	S, A, M, Co	–	–	100	T2	N1	IIb	*N* > 50
G3P3	50–60	^*^IDC	Luminal B Her negativo	DCIS-IDC	3	3	3	S, C, Co	++++	++	65	T1c	N0	I	*N* > 50
G3P4	60–70	^*^IDC	Her2	DCIS-IDC	2	2	3	S, C	–	–	36	T1a	N1	IIa	*N* > 50
G3P5	30–40	^*^IDC	Luminal B Her negativo	DCIS-IDC	2	2	2	S, C	++++	+	29	T1a	N1	IIa	*N* > 50
G3P6	50–60	^*^IDC	Luminal B Her positivo	DCIS-IDC	3	3	N	S, C, Co	++++	+	N	T1a	N0	I	*N* > 50

DCIS type: S – solid; M – micropapillary; C – cribriform; A – adherent; Co – comedocarcinoma

Clinical stage as defined by TNM staging [10] metastases have always been absent, so the clinical stage is only extrapolated from T and N

Myriad’s hereditary cancer tests were made according Frank et al. [11] - *N* < 50 (family history of hereditary cancer absent and breast cancer diagnosed before 50 years of age) and > 50 (family history of hereditary cancer absent and breast cancer diagnosed after 50 years of age)

Histological grade was obtained according Lakhani et al. [12]. For ER and PR: stained slides were examined as follows: “negative” (−) - absence of brown precipitate in cells; the positive samples were labeled as (+) [if there were a few (< 10%) scattered cells with precipitate]; (++) [for large areas (10–50%) of positivity] and (+++) [designated 50 to 100% positivity]

*IDC* invasive ductal carcinoma

*N* not available or absent

*DCIS-IDC* IDC with in-situ component

*TN* triple negative

*DCIS* ductal carcinoma in situ

*ER* estrogen receptor

*PR*- progesterone receptor

*T* tumor

*N* regional lymph node

### RNA extraction

Manual microdissection of epithelial cells was performed isolating the area with, at least, 70% of tumor cells. The DCIS-IDC samples were microdissected for both tissues.

Sample naming is as follows: non-neoplastic breast epithelium - control; pure IDC - IDC_pure_; pure DCIS - DCIS_pure_; IDC of DCIS-IDC group - IDC_comp_ and DCIS of DCIS-IDC group - DCIS_comp_.

RNA was isolated by *RecoverAll™ Total Nucleic Acid Isolation Kit* (Ambion/Life Sciences, Carlsbad, California, USA), according to manufacturer’s protocols. RNAs were quantified using NanoDrop (ThermoFisher, Waltham, Massachusetts, USA) and Qubit RNA HS Assay kit (ThermoFisher).

### Gene expression analysis

Multiplex gene expression analyses were performed at the Molecular Oncology Research Center-Barretos Cancer Hospital by *nCounter® PanCancer Pathways panel* (NanoString Technologies™, Seattle, Washington, USA), which allows the evaluation of 770 genes (730 cancer-related human genes, being 124 driver genes and 606 genes from 13 cancer-associated canonical pathways, and 40 as internal reference loci). An average of 100 ng of RNA was used for hybridization. The system analyses for gene expression digital quantification used was the *nCounter® SPRINT Profiler* (NanoString Technologies™).

### Data analysis

Raw counts expression was analyzed using the *nSolver™ Analysis Software* (NanoString Technologies™). Two-by-two comparisons were performed and differentially expressed genes (DEGs) were selected using expression levels *p*-value ≤0.01. Comparisons between the noninvasive group (control and DCIS_pure_), and the invasive group (IDC_pure_, DCIS_comp_, and IDC_comp_) were performed. A heatmap comparing the 3 tissues (control, DCIS_pure_, and IDC_pure_) was made in *nSolver™*, and a Venn diagram was constructed to select genes of interest. Gene enrichment analyses were performed by *FunRich Functional Enrichment Analysis Tool* [14], using the Gene Ontology database. Interaction network analyses were also performed at the *FunRich* using FunRich database. The UALCAN [15] was used to evaluate gene expression in BC stages available at The Cancer Genome Atlas (TCGA) database.

## Results

### Putative genes involved in DCIS progression

Eleven comparisons were made two-by-two to obtain the DEGs (*p*-value ≤0.01) (see Additional file 1: Tables S1-S11)**.** Between control and tumor tissues, the greatest differential expression was observed between DCIS_pure_ and control (123 DEGs - 72 downregulated), and the lowest, between control and IDC_pure_ (66 DEGs - 46 downregulated).

Additional file 2 Figure S1. shows the comparison of gene expression between control, DCIS_pure_, and IDC_pure_. Statistically, the invasive tissue exhibited a more similar profile to control than to the in-situ lesions. DCIS_comp_ gene expression retains more similarities with IDC_pure_ (2 DEGs), than with DCIS_pure_ (6 DEGs) and has a lower similarity with the control (104 DEGs) (see Additional file 2: Figure S1 and Additional file 1: Tables S5, S6, and S8), which suggests progressive molecular alterations from DCIS_pure_ to the IDC passing through DCIS_comp._

Among the 6 DEGs found between DCIS_pure_ and DCIS_comp_ (*FGF2*, *GAS1*, *IBSP*, *LAMC3*, *MAP3K8*, and *SFRP1*), only *IBSP* is downregulated in noninvasive lesions (Table 2).

**Table 2:** DEGs between DCIS_pure_ and DCIS_comp_ and between invasive and noninvasive groups

DEGs		Gene	t statistic	*p* value	FC^a^
DCIS_pure_ vs DCIS_comp_	Downregulated in DCIS_pure_	*IBSP*	−3.86	8,00E-03	−3.4
	Upregulated in DCIS_pure_	***FGF2***	**4.16**	**4,00E-03**	**1.5**
		***GAS1***	**3.6**	**7,00E-03**	**2.67**
		*LAMC3*	4.11	2,00E-03	2.06
		*MAP3K8*	3.49	8,00E-03	1.91
		***SFRP1***	**4.75**	**1,00E-03**	**2,61**
Noninvasive vs invasive group	Downregulated in noninvasive group	*ARID2*	−3.42	4,00E-03	−1.52
		*BCL2L1*	−3.17	6,00E-03	−1.7
		*BMP8A*	−4.11	4,00E-04	−2.28
		*CCNB1*	−3.12	5,00E-03	−1.95
		*CDC25C*	−3.16	4,00E-03	−1.91
		*OSM*	−3.11	5,00E-03	−2.23
		*UTY*	−3.1	5,00E-03	−1.97
		*WHSC1*	−3.26	6,00E-03	−1.46
	Upregulated in noninvasive group	*AXIN2*	3	8,00E-03	2.14
		*CNTFR*	2.95	9,00E-03	2.4
		*COL6A6*	3.26	7,00E-03	3.75
		*DKK1*	2.79	1,00E-02	2.02
		*DTX1*	2.82	9,00E-03	1.76
		*EFNA5*	3.21	4,00E-03	1.71
		*FGF10*	3.26	3,00E-03	3.03
		***FGF2***	**3.73**	**2,00E-03**	**2.91**
		*FGF7*	3.71	1,00E-03	2.72
		*FOS*	2.8	1,00E-02	2.6
		*FZD7*	3.3	4,00E-03	2.09
		***GAS1***	**3.67**	**1,00E-03**	**2.5**
		*GLI3*	3.11	5,00E-03	1.73
		*GRIA3*	3.7	2,00E-03	2.88
		*IGF1*	4.25	3,00E-04	2.72
		*IRS1*	3.28	6,00E-03	2.05
		*ITGA9*	3.55	2,00E-03	2.04
		*ITGB8*	3.47	2,00E-03	2.83
		*JAK1*	4.45	1,00E-04	1.32
		*JUN*	3.19	7,00E-03	2.51
		*KLF4*	3.78	1,00E-03	2.4
		*LAMB3*	2.9	1,00E-02	2.47
		*LAMC2*	2.9	1,00E-02	2.14
		*LEPR*	4.43	3,00E-04	3.08
		*LIFR*	3	7,00E-03	2.16
		*MAP3K8*	2.98	8,00E-03	1.56
		*MET*	4.09	4,00E-04	1.99
		*NGFR*	3.48	2,00E-03	2.49
		*NTRK2*	3.32	4,00E-03	4.17
		*PDGFRA*	3.85	1,00E-03	2.01
		*PLD1*	4.14	3,00E-04	1.81
		*PRKCA*	3.7	1,00E-03	1.89
		*PROM1*	2.89	9,00E-03	3.68
		*RELN*	3.05	9,00E-03	3.25
		***SFRP1***	**4.01**	**5,00E-04**	**5.79**
		*SOX17*	3.84	8,00E-04	2.41
		*SOX9*	3.01	6,00E-03	2.63
		*SRPY1*	3.73	1,00E-03	2.15
		*SRPY2*	3.47	3,00E-03	2.16
		*TCF7L1*	3.23	4,00E-03	2.15
		*TGFBR2*	3.9	7,00E-04	2.24
		*THEM4*	2.86	9,00E-03	1.6
		*TNN*	3.47	2,00E-03	2.64
		*TSC1*	2.83	9,00E-03	1.35
		*TSPAN7*	3.61	1,00E-03	2.4

In bold are the genes potentially involved in DCIS progression

*FC* fold change

*DEGs* differentially expressed genes

*DCIS*_*comp*_ DCIS as component

*DCIS*_*pure*_ pure DCIS

**Fig. 1:**
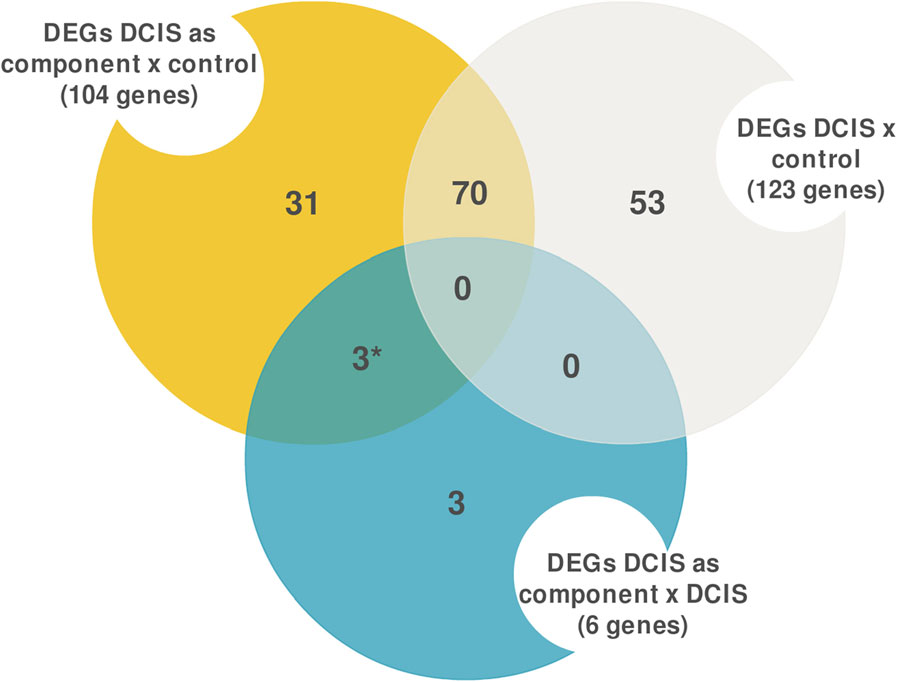
Putative genes involved in ductal carcinoma in situ (DCIS) progression. Venn diagram depicting the common and distinct genes in each comparison. Genes most likely involved in invasive capacity of pure DCIS are marked with an asterisk. DEGs - Differentially expressed genes

To verify which genes would have the greatest potential in the acquisition of invasive capacity, a Venn diagram was constructed (Fig. 1). *FGF2*, *GAS1*, and *SFRP1* are intersected between DCIS_pure_ vs DCIS_comp_ and control vs DCIS_comp_ and not present in the comparison control vs DCIS_pure_, possibly acting in the acquisition of the invasive capacity of DCIS_pure_.

The comparison between invasive and noninvasive groups shows 53 DEGs, being 8 upregulated and 45 downregulated in the invasive group (Table 2). Four of the downregulated genes in the invasive group were also differentially expressed between DCIS_pure_ and DCIS_comp_ and the genes most probably involved in the DCIS progression are among them (*FGF2*, *GAS1*, and *SFRP1*) (Table 2).

### Gene functional analysis

**Fig. 2:**
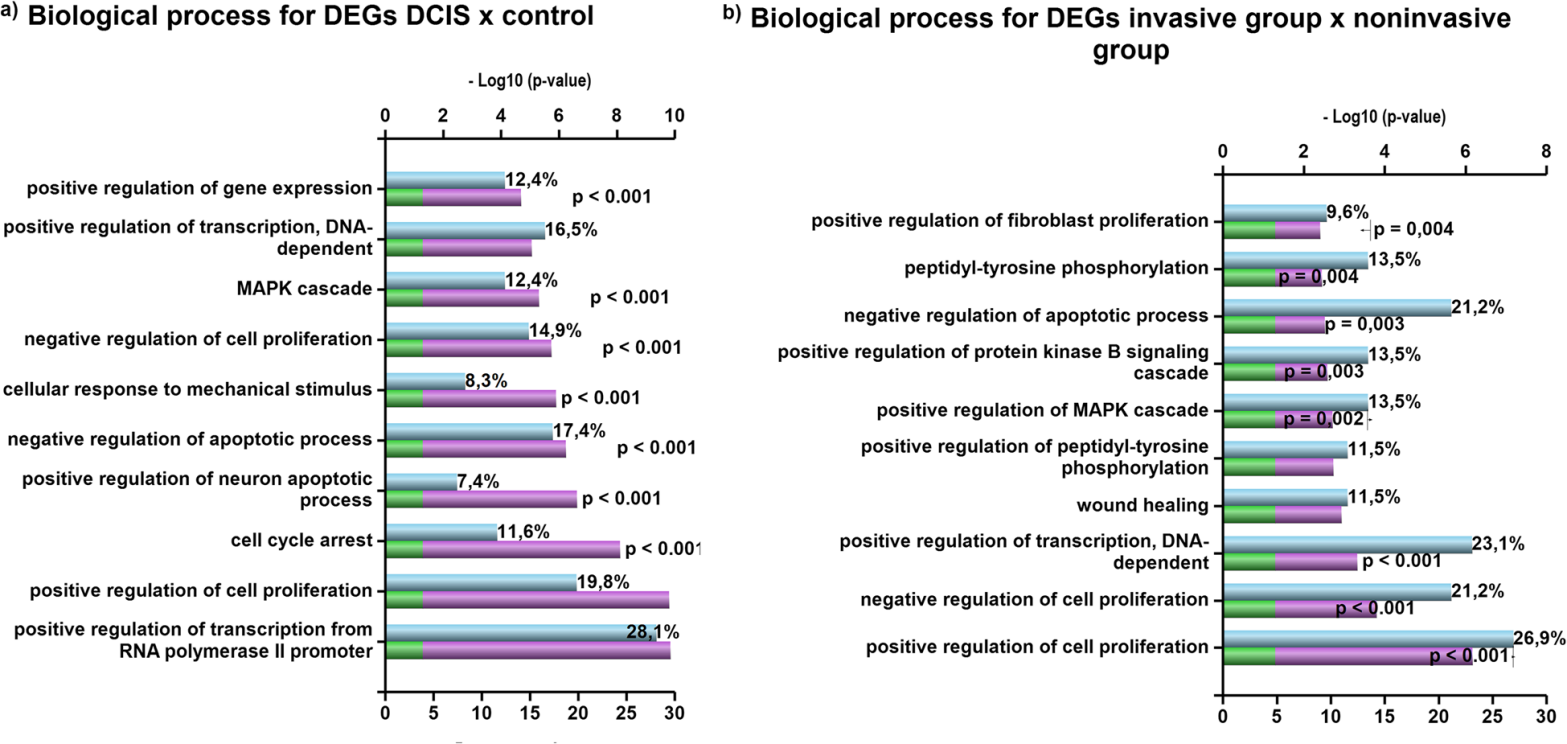
Top 10 Biological Processes for differentially expressed genes. Comparisons are between: **a** Control vs DCIS and **b** noninvasive vs invasive groups. Gene enrichment analyses were performed by *FunRich Functional Enrichment Analysis Tool*, using the Gene Ontology database

Enrichment analysis showed that the main biological processes altered between control and DCIS_pure_ (adjusted *p-*value ≤0.01) are related to gene expression regulation, cell proliferation and cell cycle arrest (Fig. 2a). Comparing invasive and noninvasive groups, the largest changes were seen in cell proliferation and transcription regulation (adjusted *p-*value ≤0.01) (Fig. 2b). To verify differences between genes potentially involved in DCIS progression (*FGF2*, *GAS1*, and *SFRP1*) and other 3 DEGs of DCIS_pure_ vs DCIS_comp_ (*LAMC3, MAP3K8*, and *IBSP)*, enrichment was done separately. In the first analysis, the most altered processes were regulation of angiogenesis, somatic stem cell maintenance, growth factor-dependent regulation of satellite cell proliferation and positive regulation of cell fate (*p-*value ≤0.01) (see Additional file 3: Table S12). For the latter ones, there were more changes in the extracellular matrix organization, differentiation cell morphogenesis and cell adhesion (*p-v*alue ≤ 0.01) (see Additional file 3: Table S12).

Protein-protein interaction (PPI) networks of the 6 DEGs of DCIS_pure_ vs DCIS_comp_ are shown in Additional file 4: Figure S2**.** In Additional file 4: Figure S2a, all interactions are shown and in Additional file 4: Figure S2b only the 107 statistically significant interactions were left in the PPI snapshot, showing 3 out of 6 genes (*p-*value ≤0.01).

**Fig. 3:**
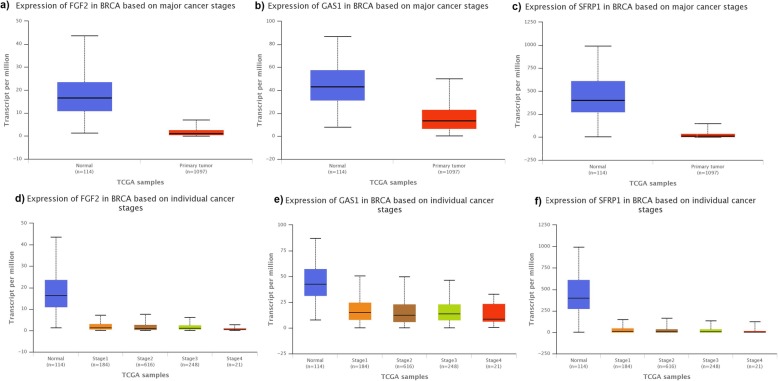
Comparisons of *FGF2*, *GAS1*, and *SFRP1* expressions. Comparisons are made between normal tissue and primary tumor (first tumor in the body) and among breast cancer (BC) progression stages (Stages 1–4). This data was generated online in UALCAN website based on The Cancer Genome Atlas database (TCGA). **a** Expression of *FGF2* in normal tissue and primary tumor. **b** Expression of *GAS1* in normal tissue and primary tumor. **c** Expression of *SFRP1* in normal tissue and primary tumor. **d** Expression of *FGF2* in BC stages. **e** Expression of *GAS1* in BC stages. **f** Expression of *SFRP1* in BC stages

Evaluation of gene expression in normal tissue and BC stages was made for 3 genes potentially involved in DCIS progression (*FGF2*, *GAS1*, and *SFRP1*) using the TCGA database (Fig. 3). The downregulation correlate with earlier stages, which corroborates our results when comparing DCIS_pure_ vs DCIS_comp_.

## Discussion

Six DEGs were found in DCIS_pure_ vs DCIS_comp_, being 3 of them also differentially expressed between control and DCIS_comp_, but not between control and DCIS_pure_. The same 3 genes (*FGF2*, *GAS1*, and *SFRP1)* showed distinct gene expression profiles between noninvasive and invasive groups. Thus, suggesting their involvement in DCIS progression.

Interestingly, the in-situ stage (DCIS_pure)_ has more molecular differences with control than the invasive stage (IDC_pure_). However, considering that IDC is the most advanced stage in progression and morphology, we expected greater molecular changes in reference to non-neoplastic tissue. Our result is probably due to early acquisition of tumor enabling features, which are later followed by minor ones [4].

DCIS_comp_ and IDC_comp_ of patients with DCIS-IDC do not have DEGs between them and are more like IDC_pure_ than control. Initial gene expression changes may remain necessary in DCIS-IDC since acquisition of invasive potential has not yet been completed in all cells. Also, as suggested by Muggerud et al. [16] and Hu et al. [17] many processes involved in DCIS progression may be expression changes in the tumor microenvironment, and not only in tumor cells [18].

The 3 DEGs more likely involved in DCIS progression were *FGF2*, *GAS1*, and *SFPR1,* all downregulated in DCIS_comp_. This fact suggests that progression from DCIS_pure_ to DCIS_comp_ may use silencing mechanisms more often than activating ones.

When comparing DEGs between control and DCIS_pure_, 31% are driver genes, whereas none of the genes that may be involved in DCIS progression or DEGs between DCIS_pure_ and IDC_pure_ is driver genes, suggesting that major alterations occur at the beginning of carcinogenesis and not at the end.

In the analysis of invasive vs noninvasive groups, *FGF2*, *GAS1,* and *SFPR1* were downregulated in the invasive group. Epigenetic alterations may contribute to BC progression by transcriptionally silencing specific tumor suppressor genes [19, 20], which could explain the loss of expression that we observed.

The expression of *FGF2* was lower in BC when compared to normal tissues [21]. In vitro assays have demonstrated a potent inhibitory effect of *FGF2* on BC cells, possibly involving MAPK cascade and cell cycle G1/S transition [22–24]. Enrichment analysis has shown statistically significant interactions between *FGF2* and MAPK pathway genes and other components of the FGF family. UALCAN analysis has shown an upregulation of *FGF2* in normal tissues, in comparison to primary BC and *FGF2* downregulation is associated with tumor progression.

According to TCGA database *GAS1* is downregulated in primary breast tumors. Hedgehog (Hh) signaling has been suggested as a critical determinant of tumor progression [25–28]. A progressive increase of Hh expression and Hh pathway activation has been observed from control, DCIS, DCIS with microinvasion and to IDC [29, 30]. GAS1 protein binds Sonic hedgehog (SHH), one of three Hh proteins, and may inhibit Hh signaling [31, 32]. The interaction of *GAS1* with *SHH* was observed but was not statistically significant.

*SFRP1* gene is a negative regulator of the Wnt pathway, which is aberrantly activated in BC [33–35]. Statistically significant interactions of *SFRP1* with Wnt pathway genes were seen and enrichment analysis showed a negative regulation of canonical Wnt receptor signaling pathway. *SFRP1* was downregulated in primary BC in comparison to normal tissue and in invasive lesions.

Functional analyses of *FGF2*, *GAS1* and *SFRP1* suggests a role in DCIS progression, being negative regulators of cell cycle G1/S transition, Hh signaling, and the Wnt pathway, respectively. We propose that downregulation favors DCIS progression. Unfortunately, our samples could not be divided into high and low-grade DCIS, nor could we study samples according to cancer molecular subtypes. Studying these groups separately may reveal important events in the DCIS progression.

## Conclusions

Understanding BC progression will enable the design of effective strategies for diagnosis and treatment. Progression biomarkers should be able to predict DCIS cases destined to become invasive tumors, therefore allowing for proper monitoring and avoiding overtreatment. Here, we identified 3 progression-specific candidate genes namely *FGF2*, *GAS1*, *SFRP1,* downregulated in tissues with invasive capacity. The progression from DCIS to invasive BC is a complex process, being possible that DCIS of distinct molecular phenotypes progress to invasive BC through the acquisition of distinct genetic or epigenetic hits.

## Supplementary information

**Additional file 1: Table S1.** Comparison between control and pure invasive ductal carcinoma. Differentially expressed genes are in bold. **Table S2.** Comparison between control and pure ductal carcinoma in situ. Differentially expressed genes are in bold. **Table S3.** Comparison between pure carcinoma ductal in situ and pure invasive ductal carcinoma. Differentially expressed genes are in bold. **Table S4.** Comparison between invasive ductal carcinoma of mixed lesions and pure invasive ductal carcinoma. Differentially expressed genes are in bold. **Table S5.** Comparison between ductal carcinoma in situ of mixed lesions and pure invasive ductal carcinoma. Differentially expressed genes are in bold. **Table S6.** Comparison between pure ductal carcinoma in situ and ductal carcinoma in situ of mixed lesions. Differentially expressed genes are in bold. **Table S7.** Comparison between pure ductal carcinoma in situ and invasive ductal carcinoma of mixed lesions. Differentially expressed genes are in bold. **Table S8.** Comparison between control and ductal carcinoma in situ of mixed lesions. Differentially expressed genes are in bold. **Table S9.** Comparison between pure ductal carcinoma in situ of mixed lesions and invasive ductal carcinoma of mixed lesions. Differentially expressed genes are in bold. **Table S10.** Comparison between control and invasive ductal carcinoma of mixed lesions. Differentially expressed genes are in bold. **Table S11.** Comparison between noninvasive and invasive groups. Differentially expressed genes are in bold.

**Additional file 2: Figure S1.** Hierarchical clustering of 730 genes and its gene expressions. Genes of *nCounter® PanCancer Pathways panel*. Gene expressions are in non-neoplastic (control), ductal carcinoma in situ (DCIS) and invasive ductal carcinoma (IDC) tissues. Agglomerative clustering was made in *nSolver™ Analysis Software.* Individual genes are arranged in rows and samples’ groups in columns. The color scale is shown above the figure.

**Additional file 3: Table S12.** Top 10 biological process of DEGs between DCIScomp and DCISpure and comparisons with control tissue.

**Additional file 4: Figure S2.** Snapshot of protein-protein interaction networks. Networks are made with the 6 differentially expressed genes between ductal carcinoma in situ as component (DCIS_comp_) and pure DCIS (DCIS_pure_). Interaction diagram was generated using *FunRich Functional Enrichment Analysis Tool* and FunRich database. a) Network diagram with all annotated interactions. b) Network diagram illustrating the 107 statistically significant interactions (*p*-value ≤0.01).

## Data Availability

All data generated or analyzed during this study are included in this published article [and its supplementary information files].

## Abbreviations

BC: Breast cancer
DCIS: Ductal carcinoma in situ
DEG: Differently expressed gene
FFPE: Formalin-fixed paraffin-embedded
Hh: Hedgehog
IDC: Invasive ductal carcinoma
PPI: Protein-protein interaction
QC: Quality Control
SHH: Sonic hedgehog
TCGA: The cancer genome atlas
