# Supplementary figures and images for: Potential biomarkers of ductal carcinoma in situ progression
Source: BMC Cancer. 2020 Feb 12;20:119. doi: 10.1186/s12885-020-6608-y (PMC7017577; doi:10.1186/s12885-020-6608-y)

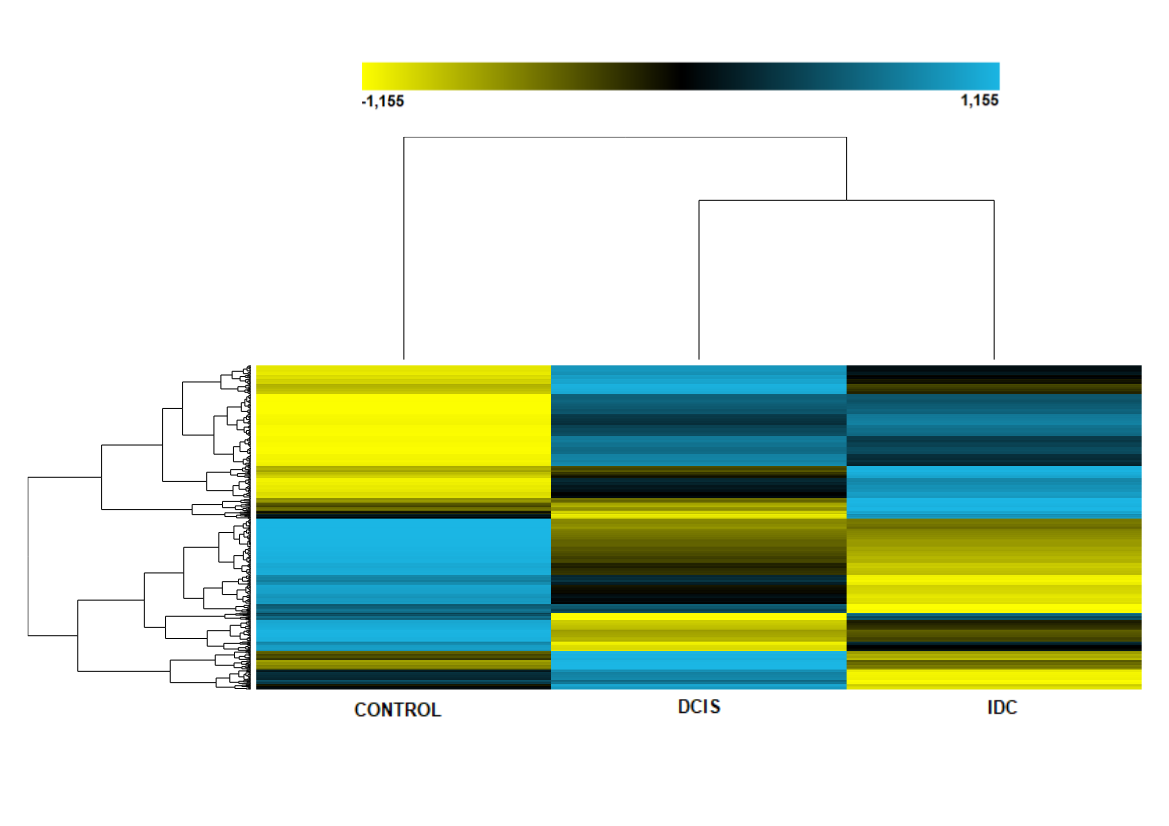

Supplement: Supplementary file 2 — Additional file 2: Figure S1. Hierarchical clustering of 730 genes and its gene expressions. Genes of nCounter® PanCancer Pathways panel. Gene expressions are in non-neoplastic (control), ductal carcinoma in situ (DCIS) and invasive ductal carcinoma (IDC) tissues. Agglomerative clustering was made in nSolver™ Analysis Software. Individual genes are arranged in rows and samples’ groups in columns. The color scale is shown above the figure. [file 12885_2020_6608_MOESM2_ESM.tiff]

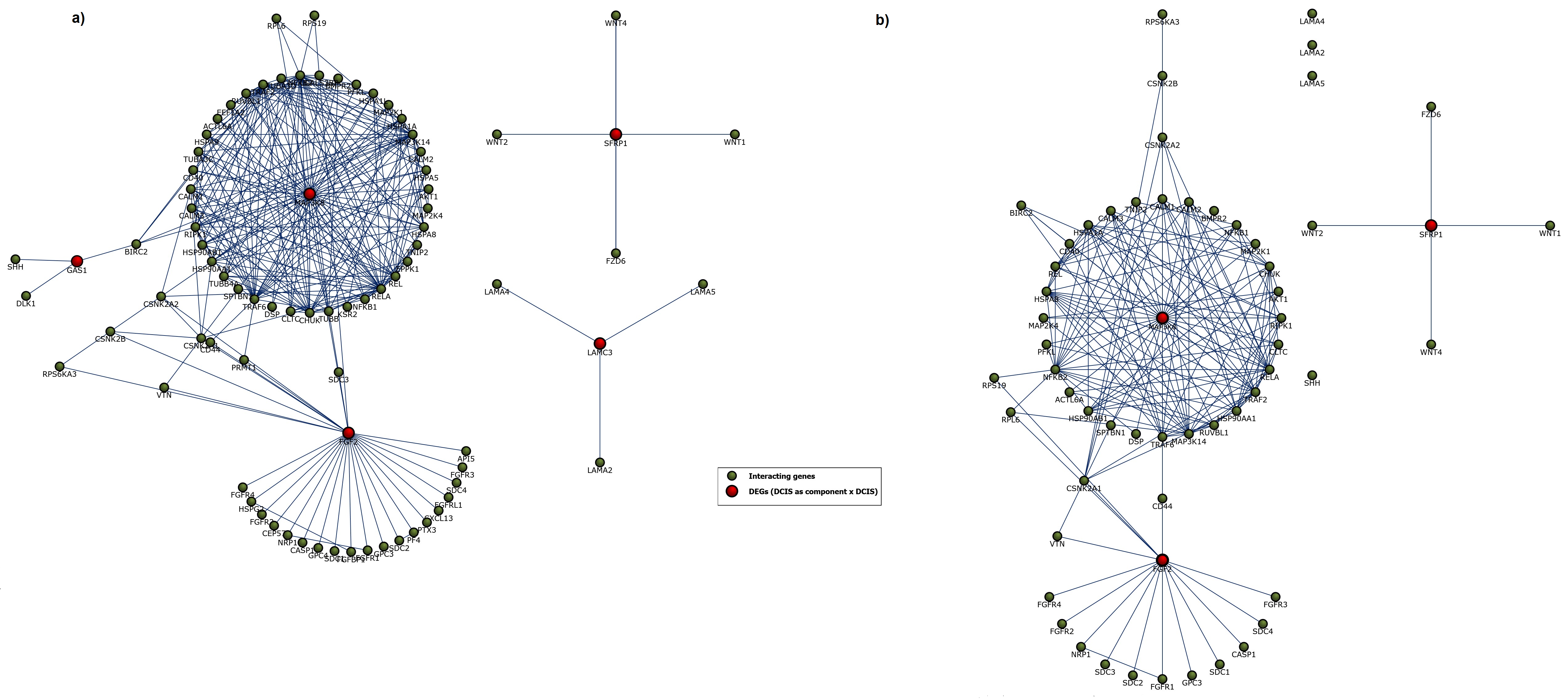

Supplement: Supplementary file 4 — Additional file 4: Figure S2. Snapshot of protein-protein interaction networks. Networks are made with the 6 differentially expressed genes between ductal carcinoma in situ as component (DCIScomp) and pure DCIS (DCISpure). Interaction diagram was generated using FunRich Functional Enrichment Analysis Tool and FunRich database. a) Network diagram with all annotated interactions. b) Network diagram illustrating the 107 statistically significant interactions (p-value ≤0.01). [file 12885_2020_6608_MOESM4_ESM.tiff]
