# Supplementary material for: Potential biomarkers of ductal carcinoma in situ progression
Source: BMC Cancer. 2020 Feb 12;20:119. doi: 10.1186/s12885-020-6608-y (PMC7017577; doi:10.1186/s12885-020-6608-y)
Supplement: Supplementary file 3 — Additional file 3: Table S12. Top 10 biological process of DEGs between DCIScomp and DCISpure and comparisons with control tissue. [file 12885_2020_6608_MOESM3_ESM.docx]

**Table S12.** Top 10 biological process of DEGs between DCIS_comp_ and DCIS_pure_ and comparisons with control tissue.

| **DCIS_pure_ vs DCIS_comp_ DEGs** | | |
| --- | --- | --- |
| **Biological Process** | **Genes in database** | ***p*-value (Ht)^a^** |
| Extracellular matrix organization | *FGF2, LAMC3, IBSP* | 2,59E-05 |
| Regulation of angiogenesis | *FGF2, SFRP1* | 4,37E-05 |
| Cellular response to growth factor stimulus | *IBSP, SFRP* | 8,87E-05 |
| Somatic stem cell maintenance | *FGF2, SFRP1* | 1,88E-04 |
| Growth factor dependent regulation of satellite cell proliferation | *FGF2* | 3,36E-04 |
| Positive regulation of cell fate specification | *FGF2* | 3,36E-04 |
| Stromal-epithelial cell signaling involved in prostate gland development | *SFRP1* | 3,36E-04 |
| Convergent extension involved in somitogenesis | *SFRP1* | 3,36E-04 |
| Negative regulation of canonical Wnt receptor signaling pathway | *SFRP1* | 3,36E-04 |
| Osteoblast differentiation | *IBSP, SFRP1* | 4,69E-04 |
| **DCIS_pure_ vs DCIS_comp_ DEGs, excluding control vs DCIS_comp_ and control vs DCIS_pure_ DEGs** | | |
| **Biological Process** | **Genes in database** | ***p*-value (Ht)** |
| Extracellular matrix organization | *LAMC3, IBSP* | 3,62E-04 |
| Cell morphogenesis involved in differentiation | *LAMC3* | 6,73E-04 |
| Cell adhesion | *LAMC3, IBSP* | 2,00E-03 |
| Astrocyte development | *LAMC3* | 2,86E-03 |
| Stress-activated MAPK cascade | *MAP3K8* | 4,54E-03 |
| Bone mineralization | *IBSP* | 6,04E-03 |
| Positive regulation of cell adhesion | *IBSP* | 7,22E-03 |
| Cellular response to growth factor stimulus | *IBSP* | 7,38E-03 |
| Activation of MAPKK activity | *MAP3K8* | 8,22E-03 |
| Stress-activated protein kinase signaling cascade | *MAP3K8* | 8,56E-03 |
| **DCIS_pure_ vs DCIS_comp_ DEGs, including control vs DCIS_comp_ and excluding control vs DCIS_pure_ DEGs** | | |
| **Biological Process** | **Genes in database** | ***p*-value (Ht)** |
| Regulation of angiogenesis | *FGF2, SFRP1* | 8,76E-06 |
| Somatic stem cell maintenance | *FGF2, SFRP1* | 3,79E-05 |
| Growth factor dependent regulation of satellite cell proliferation | *FGF2* | 1,68E-04 |
| Positive regulation of cell fate specification | *FGF2* | 1,68E-04 |
| Stromal-epithelial cell signaling involved in prostate gland development | *SFRP1* | 1,68E-04 |
| Convergent extension involved in somitogenesis | *SFRP1* | 1,68E-04 |
| Negative regulation of canonical Wnt receptor signaling pathway involved in controlling type B pancreatic cell proliferation | *SFRP1* | 1,68E-04 |
| Negative regulation of bone remodeling | *SFRP1* | 3,36E-04 |
| Neural crest cell fate commitment | *SFRP1* | 3,36E-04 |
| GO:1904956 | *SFRP1* | 3,36E-04 |

Ht - hypergeometric test.

DEGs - differentially expressed genes.

DCIS_comp_ - DCIS as component.

DCIS_pure_ - pure DCIS.
